# Supplementary material for: Socioeconomic predictors of vulnerability to flood-induced displacement
Source: Nat Commun. 2025 Sep 16;16:8296. doi: 10.1038/s41467-025-64015-8 (PMC12441118; doi:10.1038/s41467-025-64015-8)
Supplement: Supplementary file 1 — Supplementary Information [file 41467_2025_64015_MOESM1_ESM.pdf]

“Socioeconomic predictors of vulnerability to flood-induced displacement”

by Benedikt Mester, Katja Frieler, Oliver Korup, Bina Desai, and Jacob Schewe
Email: jacob.schewe@pik-potsdam.de

Supplementary Information

Contents

Supplementary Figures 2
1 Vulnerability 2
2 Explained variance 5
3 Alternative feature importance rankings 6
4 Alternative outcome: Fatalities 8
5 Additional Partial Dependence Plots 9
6 Cross-correlation 10
7 Alternative displacement data 12
8 Methods: Data and analysis workflow 19
Supplementary Tables 20

Supplementary Figures

1 Vulnerability

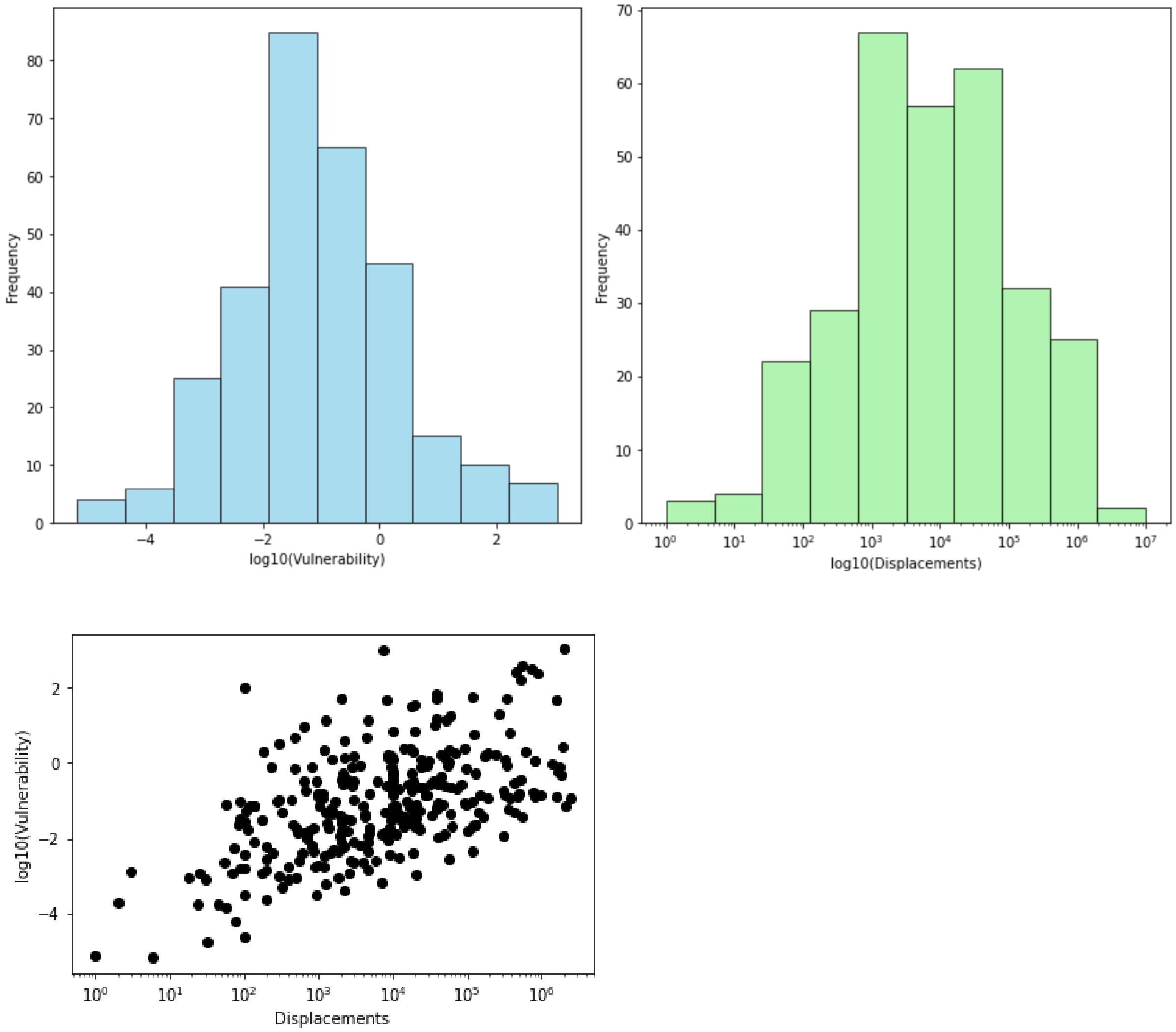

Figure S1. Distribution of displacement and vulnerability data (n=303 events). Top: Frequency distribution of log10 vulnerability ratio (left) and log10 number of displacements (right). Bottom: scatterplot of vulnerability vs. displacements.

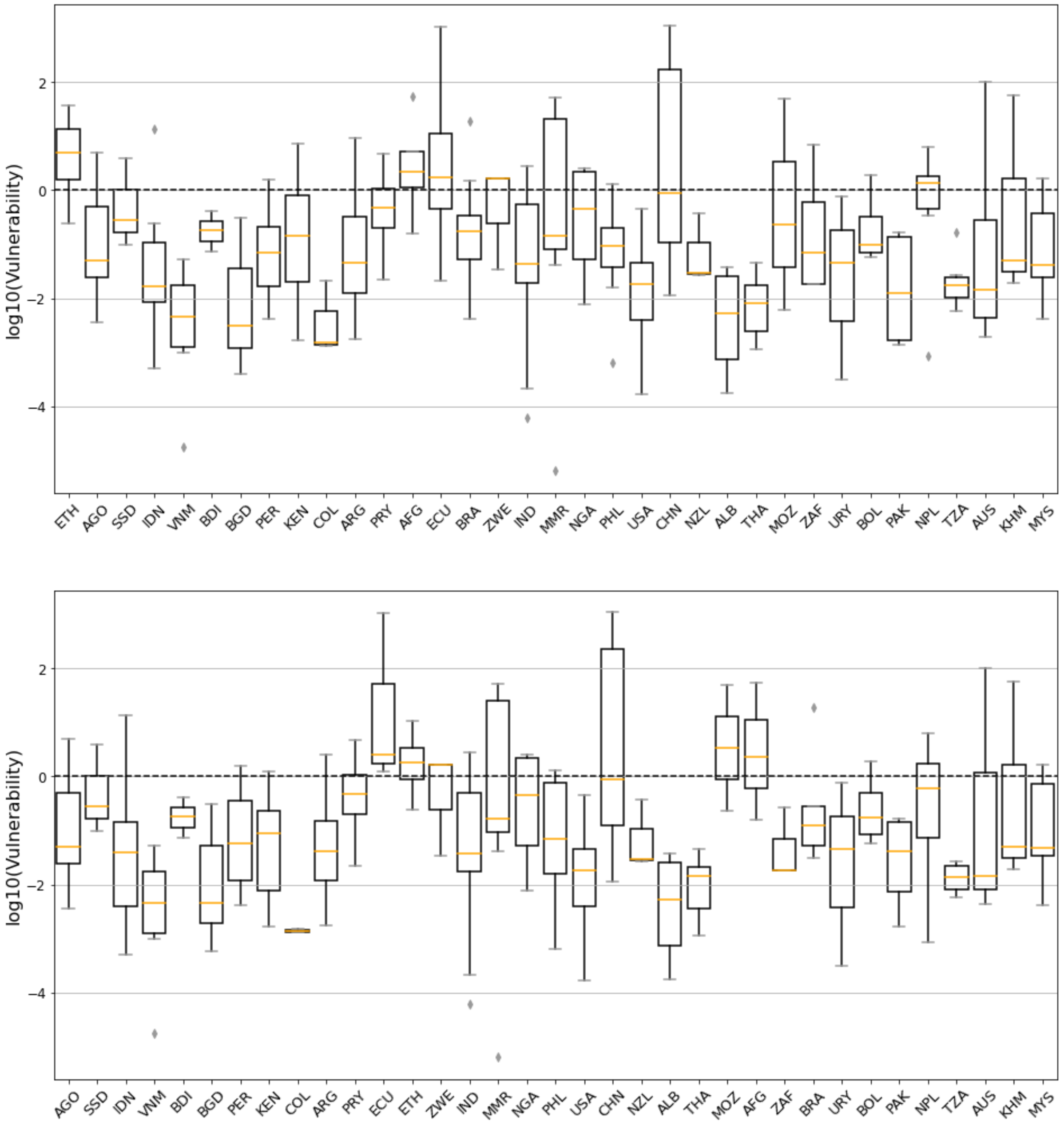

Figure S2. Spread in vulnerability for countries with at least 3 data entries. Each box represents the interquartile range of vulnerability values, with the median indicated by the line inside the box. Whiskers extend to 1.5 times the interquartile range above and below the box, and outliers are displayed as diamonds. Countries indicated by ISO3 codes. Top, 2008-2018; bottom, 2013-2018 (exluding years with lower displacement data quality).

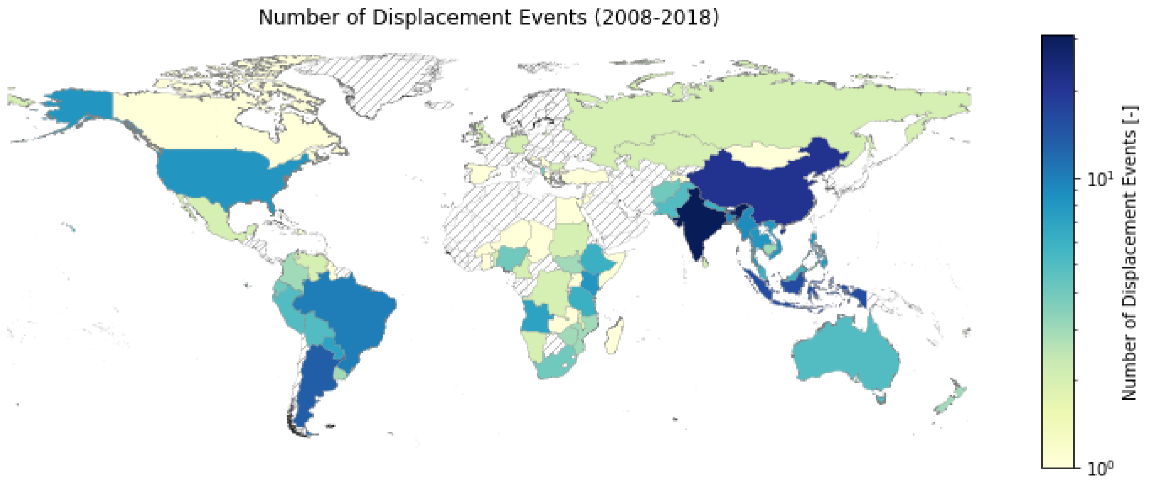

Figure S3. Number of displacement events in sample. Hatching indicates countries with no data availability. Country borders are derived from GADM (GADM, 2018).

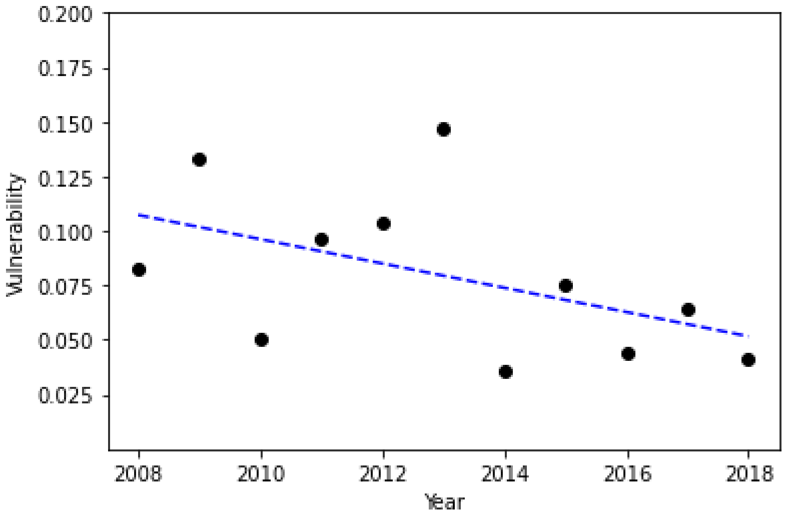

Figure S4. Global annual median of vulnerability to flood-induced displacement. Dashed line is a linear fit. The estimated slope is not significantly different from zero (Mann-Kendall trend test p=0.16).

2 Explained variance

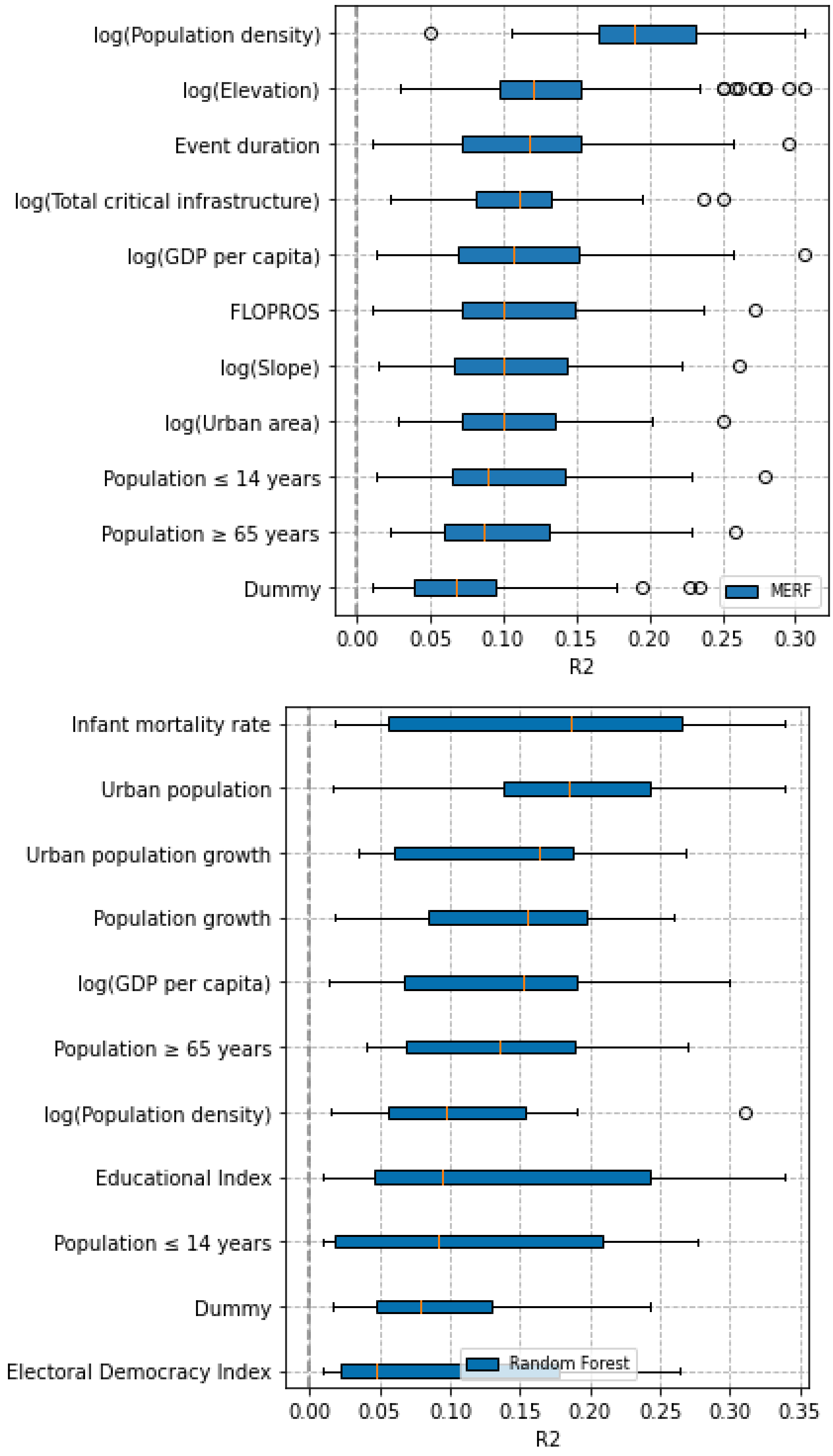

Figure S5. Explained variance for event-level (top; n=303) and country-level (bottom; n=72) models, ranked by median R^2. Results relate to the test data using leave-one-out cross-validation. Box plots as in Fig. 2 in the main paper but without indicating the effect sign. Only models with R^2 greater than 0.01 are displayed.

3 Alternative feature importance rankings

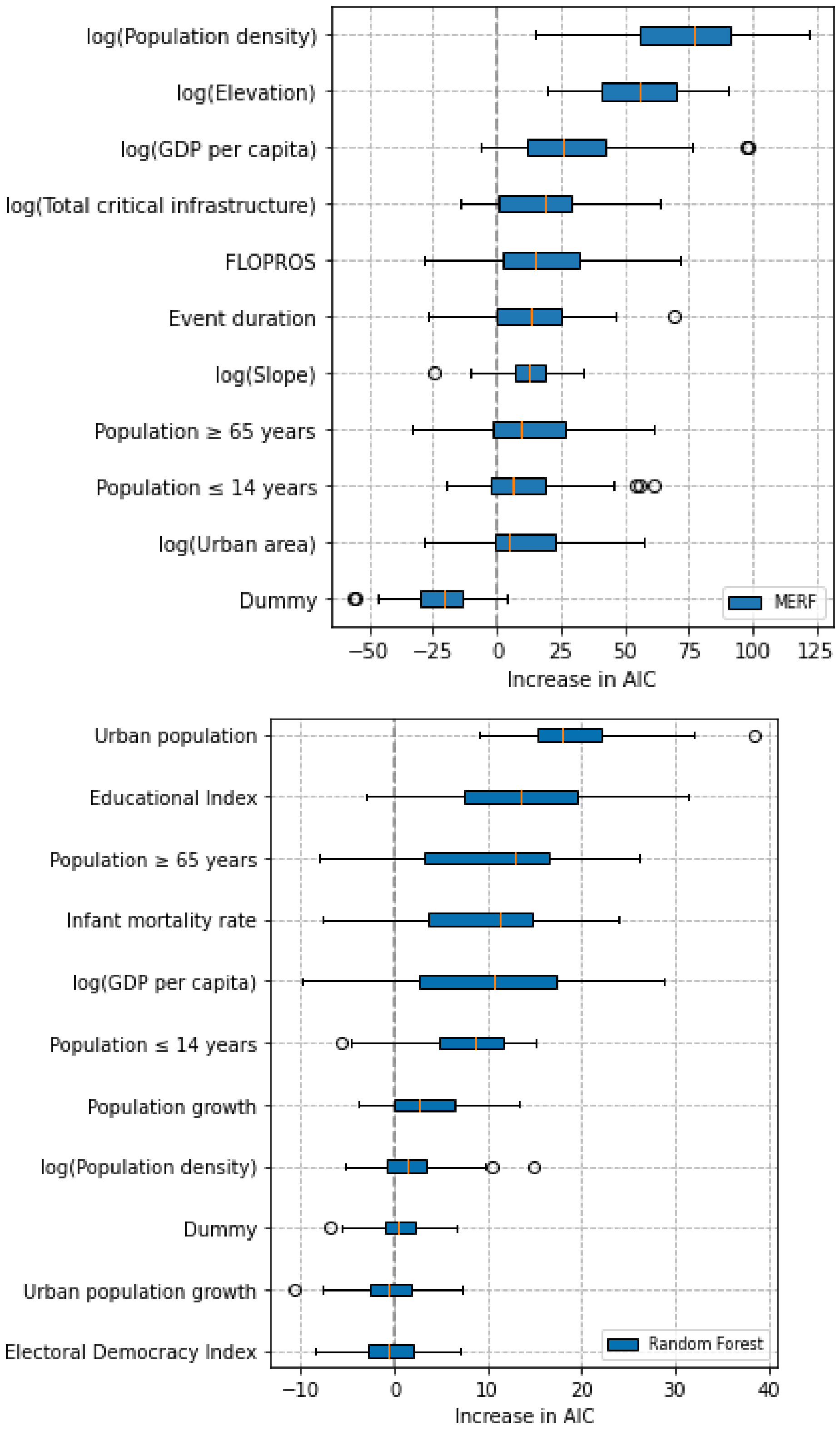

Figure S6. As Fig. 2 in the main paper, but with feature importance measured by the median increase in AIC after randomizing. Top: event-level analysis, bottom: country-level analysis.

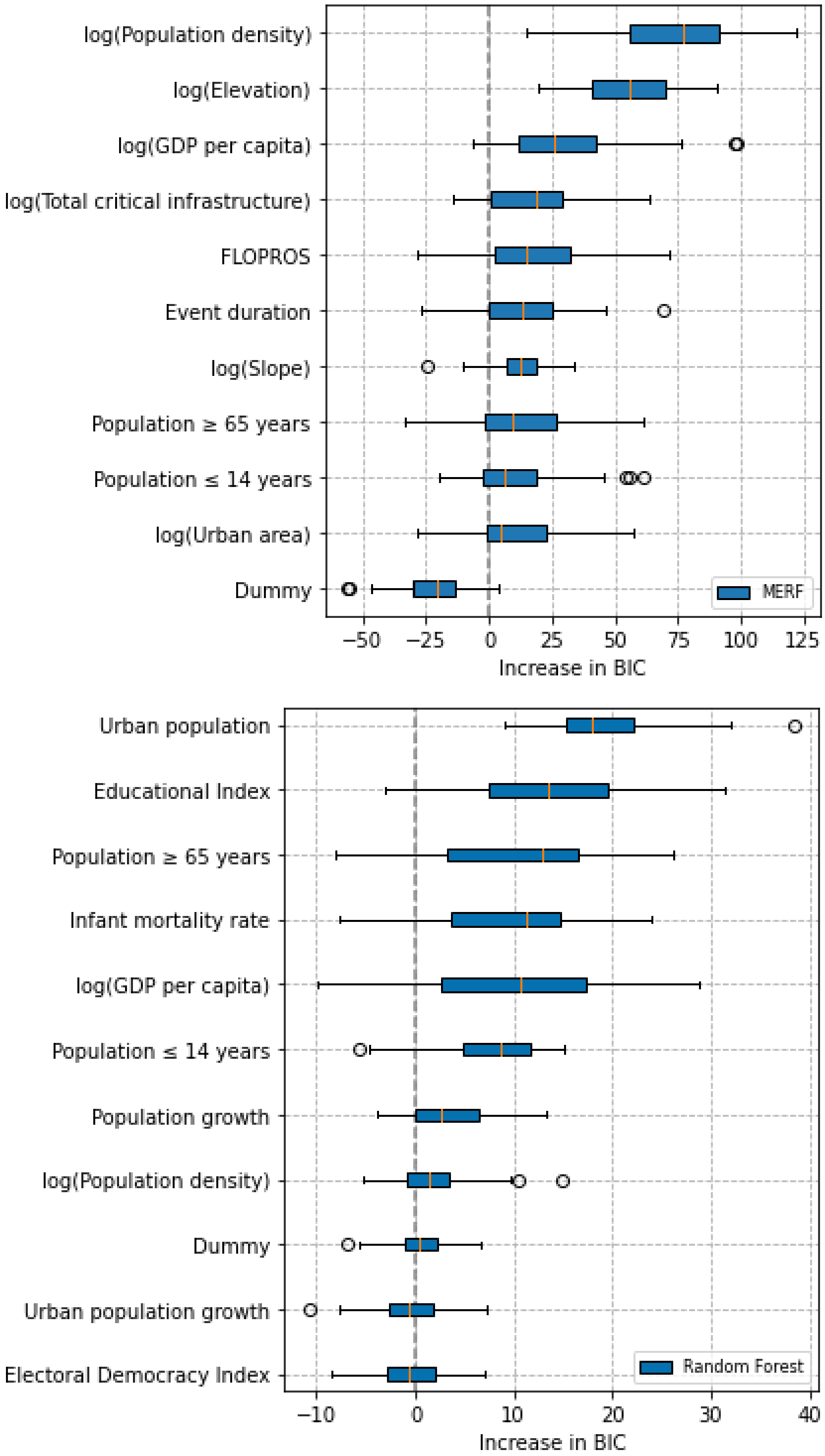

Figure S7. As Fig. 2 in the main paper, but with feature importance measured by the median increase in BIC after randomizing. Top: event-level analysis, bottom: country-level analysis.

4 Alternative outcome: Fatalities

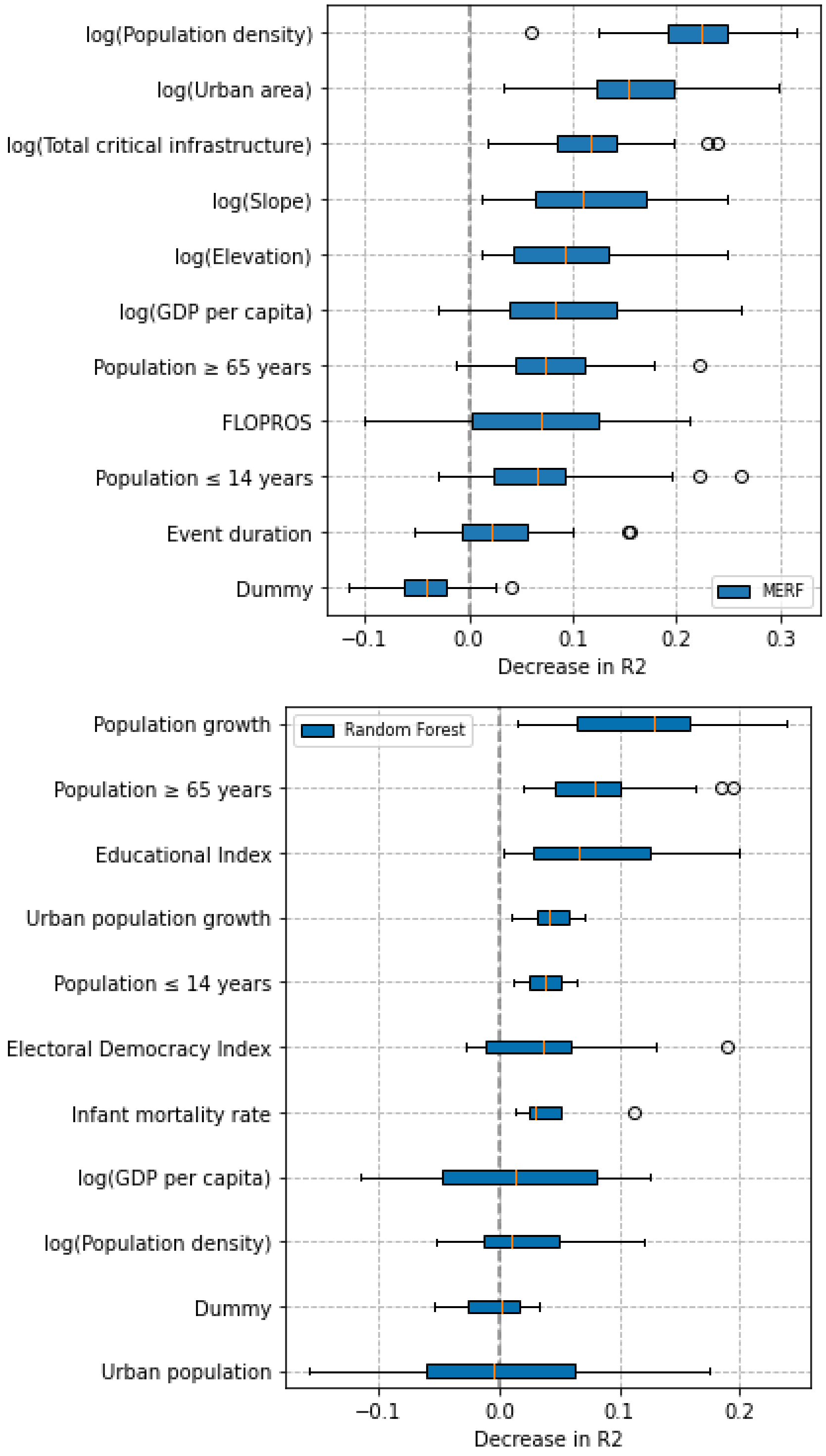

Figure S8. As Fig. 2 in the main paper but using the ratio of deaths-to-exposed as outcome variable, rather than displacements-to-exposed. The data includes 195 events in 55 countries (n=195 in top panel; n=55 in bottom panel).

5 Additional Partial Dependence Plots

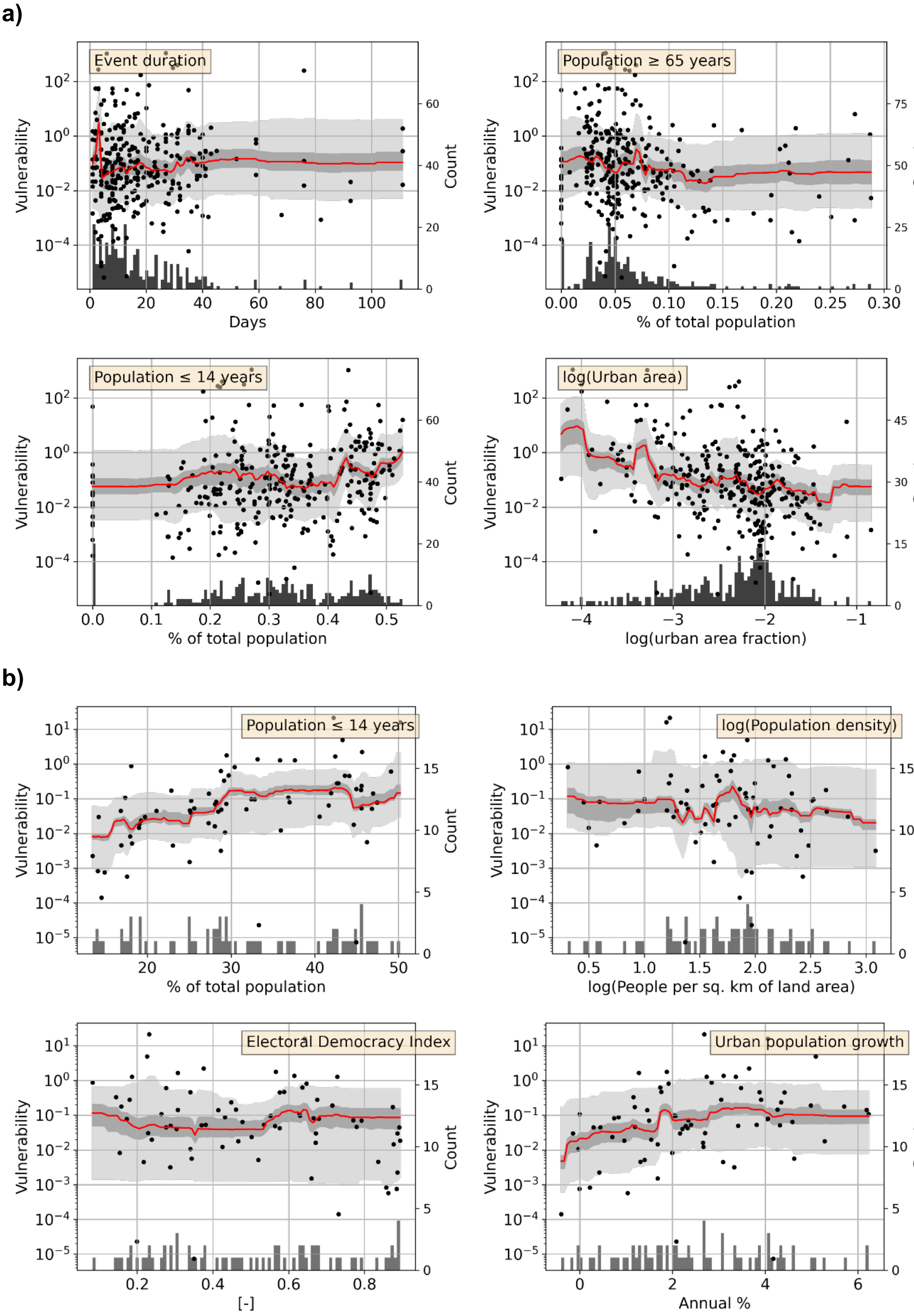

Figure S9. Partial dependence plots for predictors not shown in the main paper. a) As Fig. 3 in the main paper but for additional predictors in the event-level analysis. b) As Fig. 4 in the main paper but for additional predictors in the country-level analysis.

6 Cross-correlation

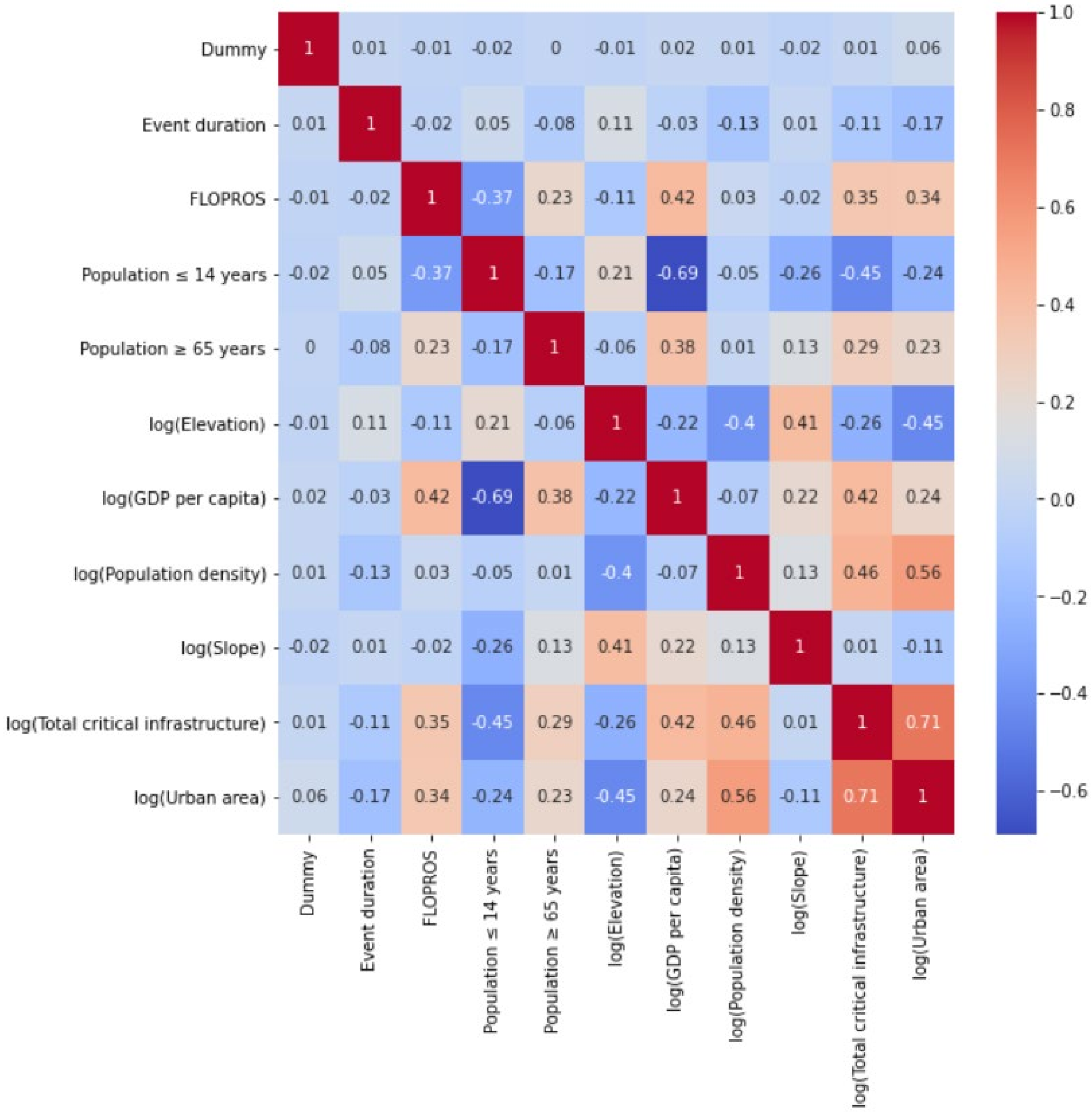

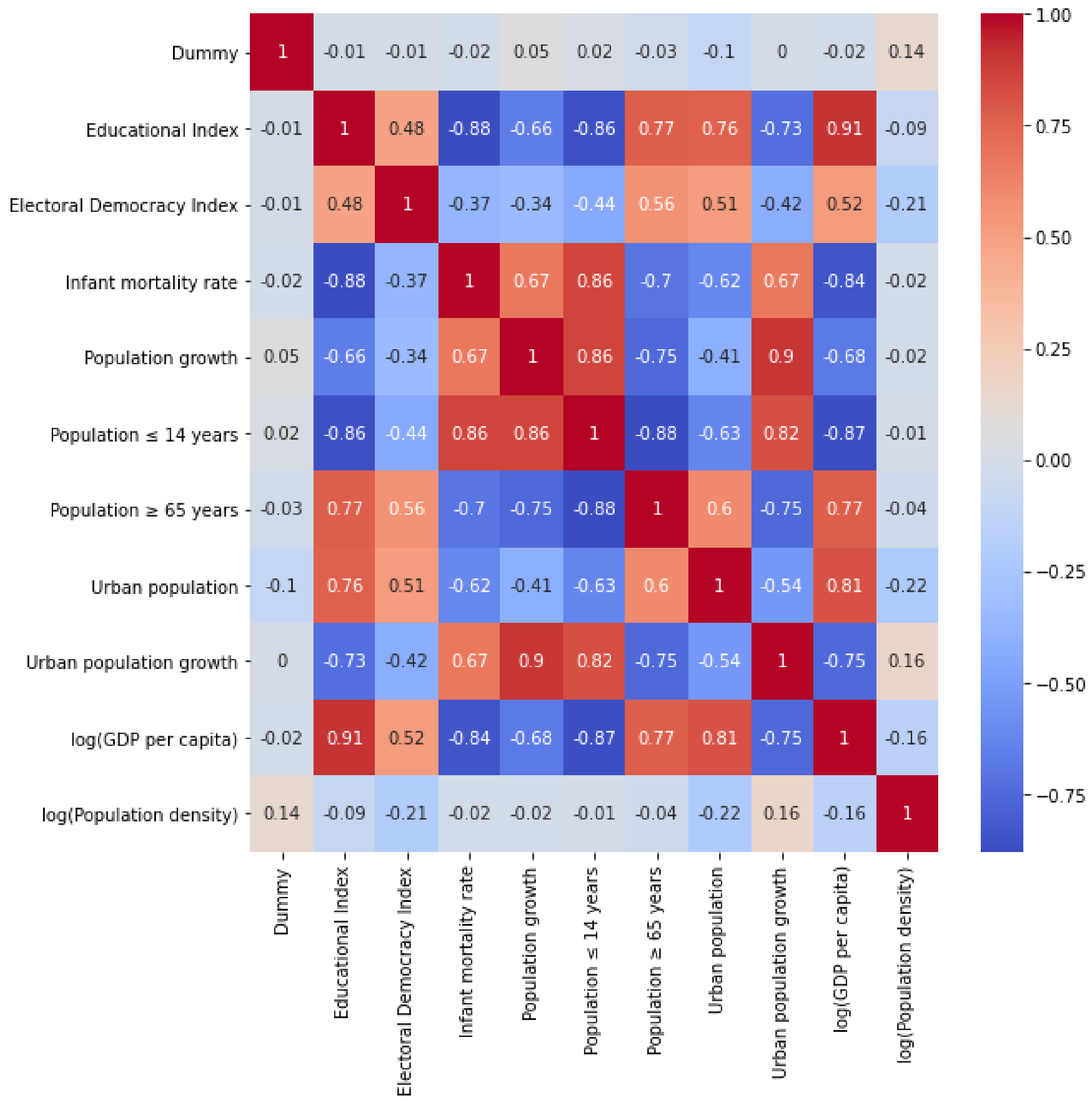

Figure S10. Pearson correlation matrix of subnational (top; n=303) and country-level (bottom; n=55) predictors. Refers only to the observations included in the main analysis, i.e. where a displacement vulnerability ratio could be calculated.

7 Alternative displacement data

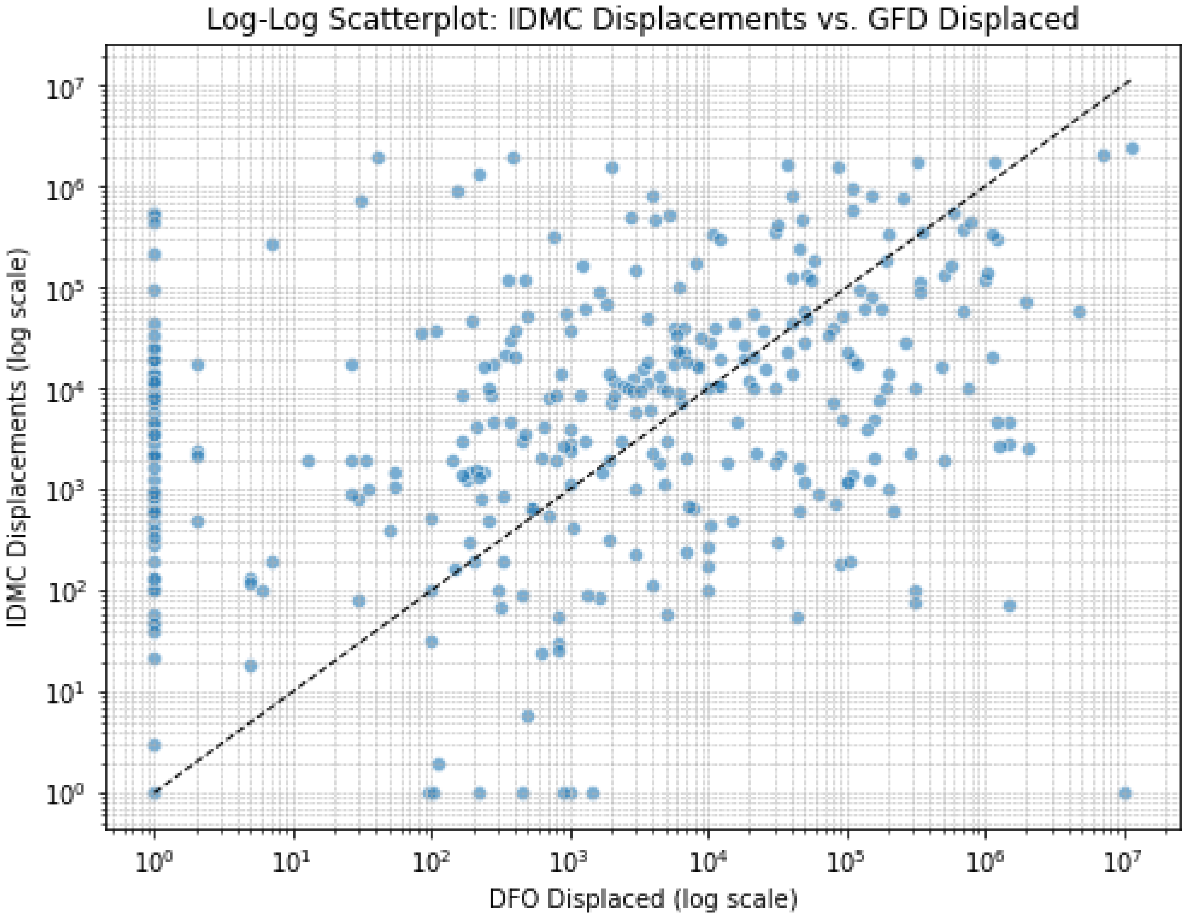

Figure S11. Comparison of displacement data from DFO and IDMC, for 335 flood events with non-zero displacements according to at least one of the sources. Pearson correlation coefficient 0.309, P-value 7.223e-09. Zero-values are displayed as ones (10^0). The figure includes all events for which an IDMC displacement record could be matched to a GFD flood event (and thus also to a DFO flood event). It includes eight events for which the IDMC displacement estimate is zero, and another 24 events for which our estimate of population exposure is zero; therefore, 32 events are included here that are not included in our main analysis. The DFO displacement estimate is zero for 52 events shown in this figure.

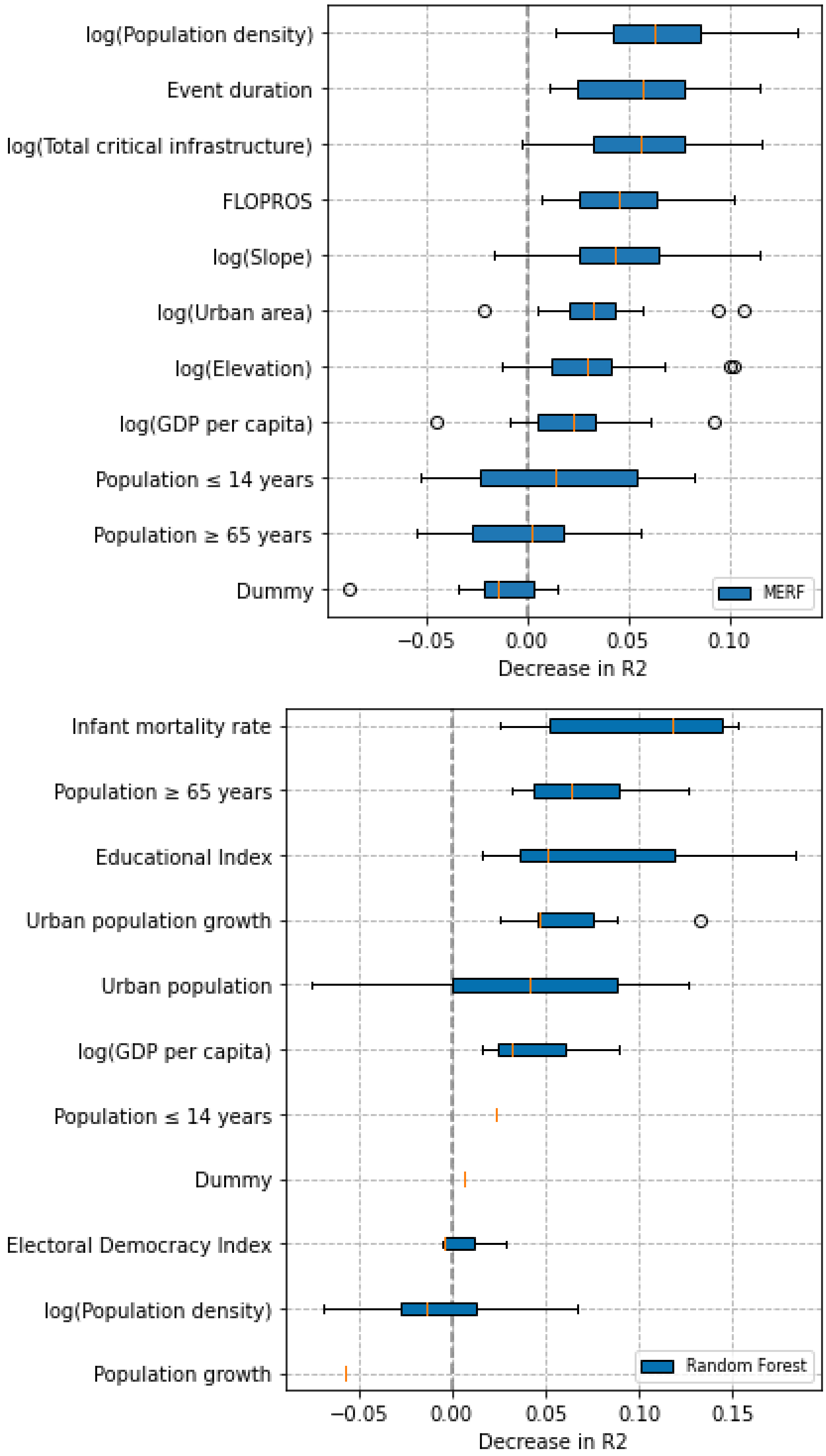

Figure S12: As Fig. 2 in the main paper but using DFO data instead of IDMC data. Includes events for which non-zero displacement estimates are available from both IDMC and DFO (n=270 events (top) in n=72 countries (bottom)).

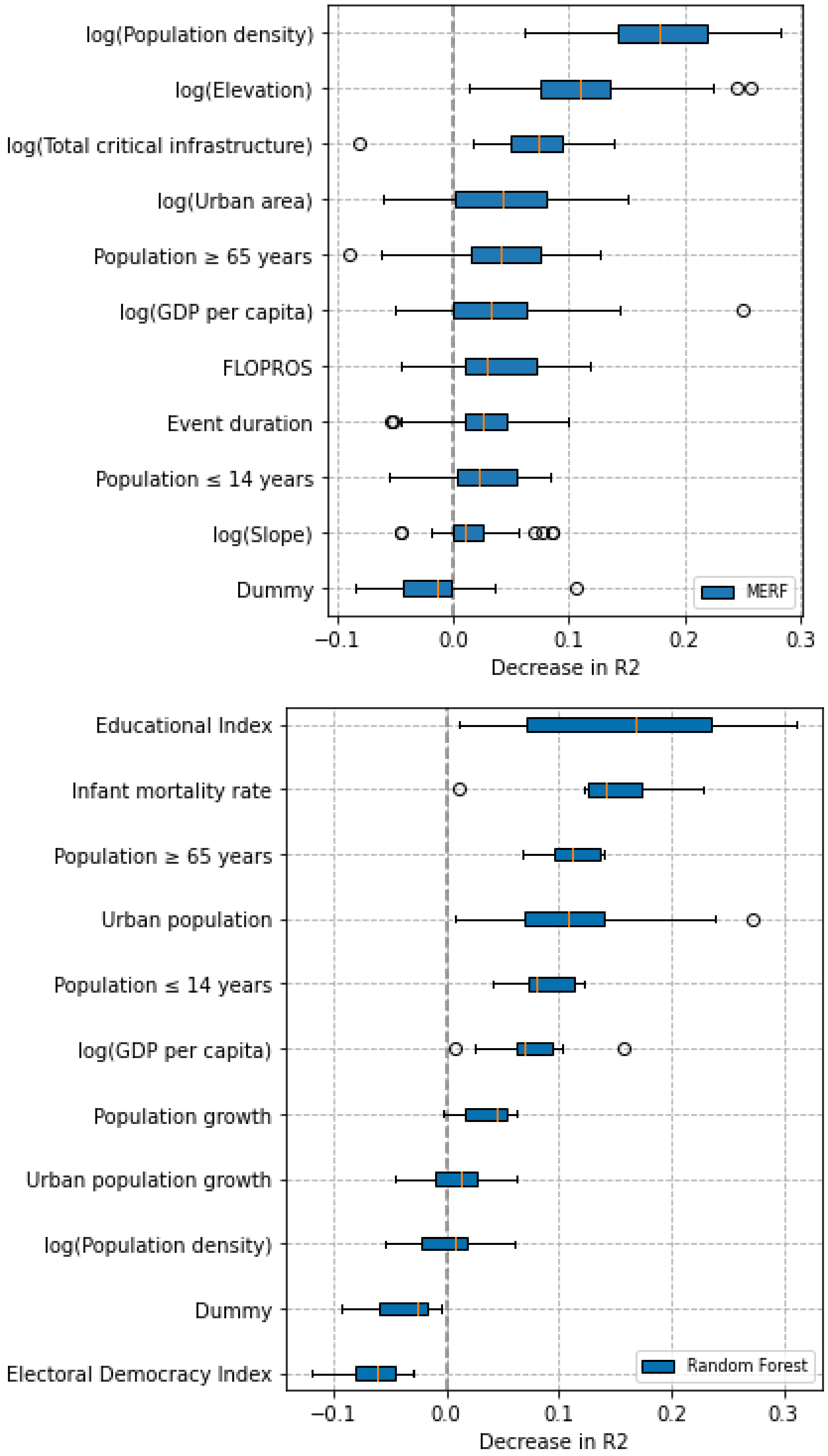

Figure S13: As Fig. 2 in the main paper (IDMC data) but only for those events for which non-zero displacement estimates are available from both IDMC and DFO. Top: n=270 events. Bottom: n=72 countries.

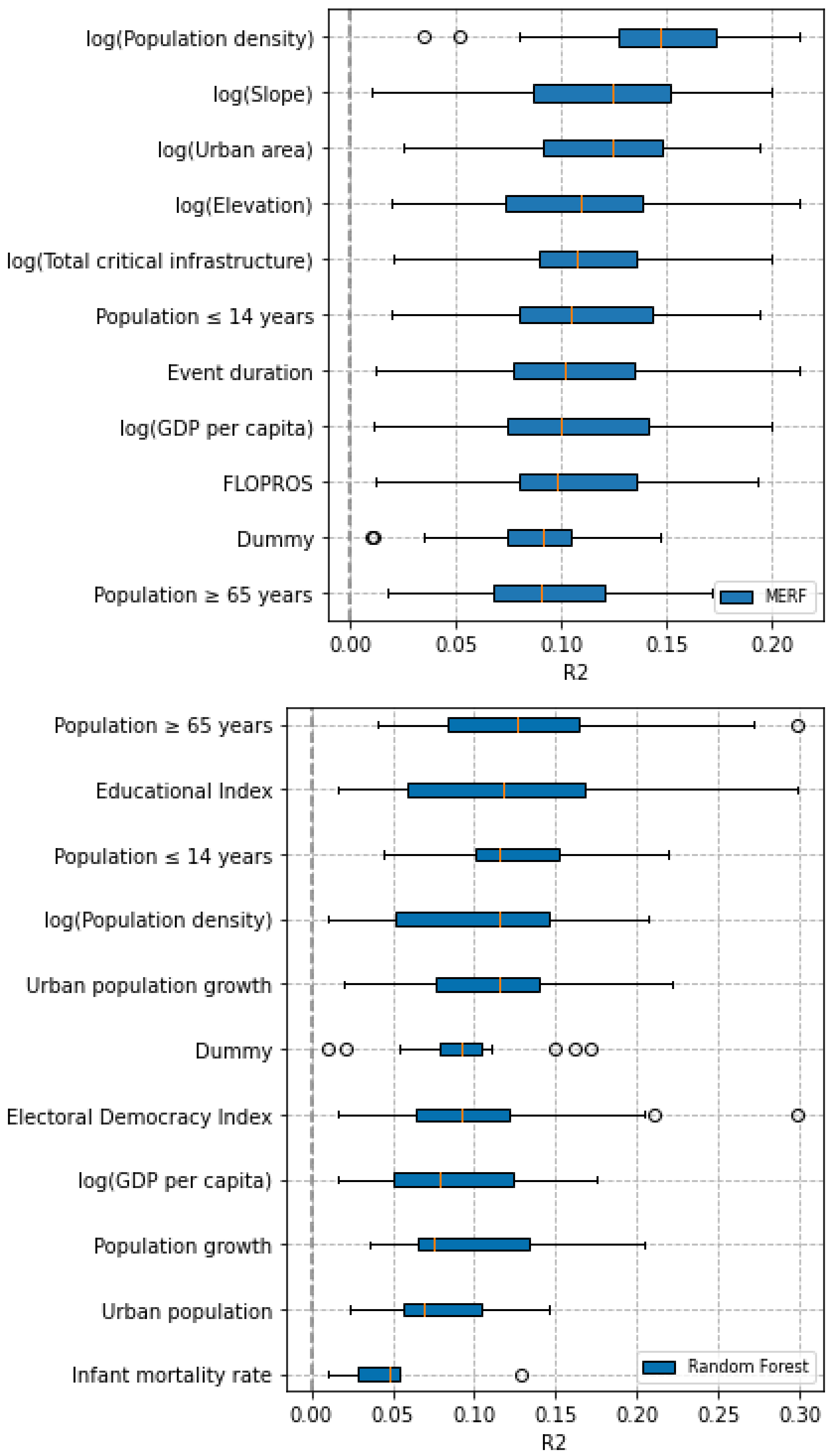

Figure S14: As Fig. S5 but using DFO displacement data instead of IDMC data, for n=678 flood events (top) in n=94 countries (bottom). The number of events is larger here than for the main analysis using IDMC data, mainly because for many of the GFD flood events a displacement record exists in DFO but no corresponding IDMC displacement record could be identified. Note that unlike IDMC records, DFO displacement records are associated with a polygon representing an approximate area affected by the flood, but not necessarily with a specific country. Therefore, only a single displacement estimate is provided for flood events affecting multiple countries. The GFD dataset originally contains

911 entries with valid polygons. After excluding entries related to Taiwan and North Korea - countries not included in the World Bank database - the total is reduced to 904. Of these, 65 entries list an "OtherCountry," indicating that more than one country was affected. For the purpose of the country-level analysis, we assign such events to the country listed in the "Country" column by DFO, and do not attempt to split displacement estimates between multiple countries, as this would require additional assumptions.

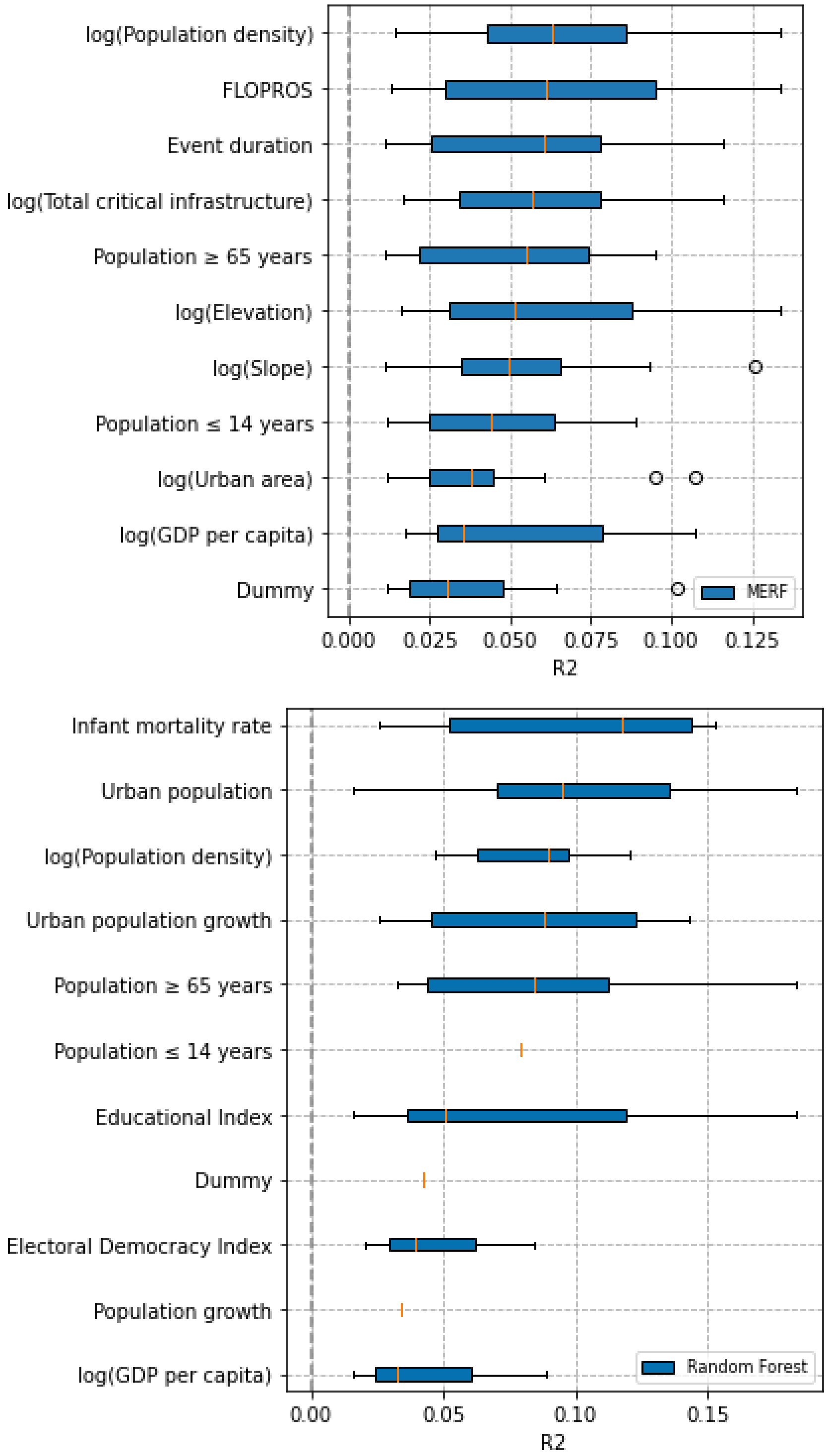

Figure S15: As Fig. S13 (DFO data) but only for those events for which both IDMC and DFO estimates are available. Top: n=270 events. Bottom: n=72 countries.

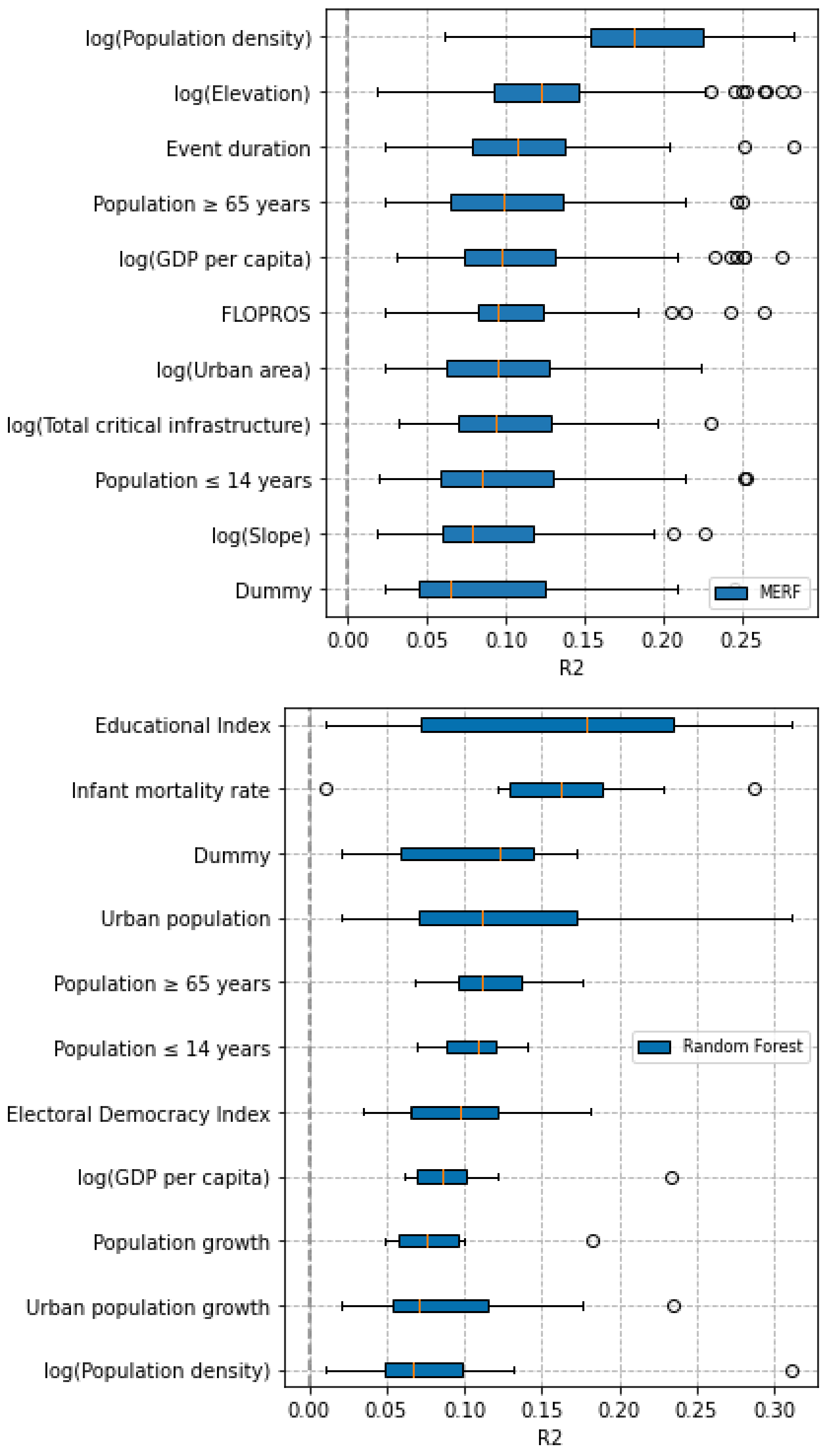

Figure S16: As Fig. S5 (IDMC data) but only for those events for which both IDMC and DFO estimates are available. Top: n=270 events. Bottom: n=72 countries.

8 Methods: Data and analysis workflow

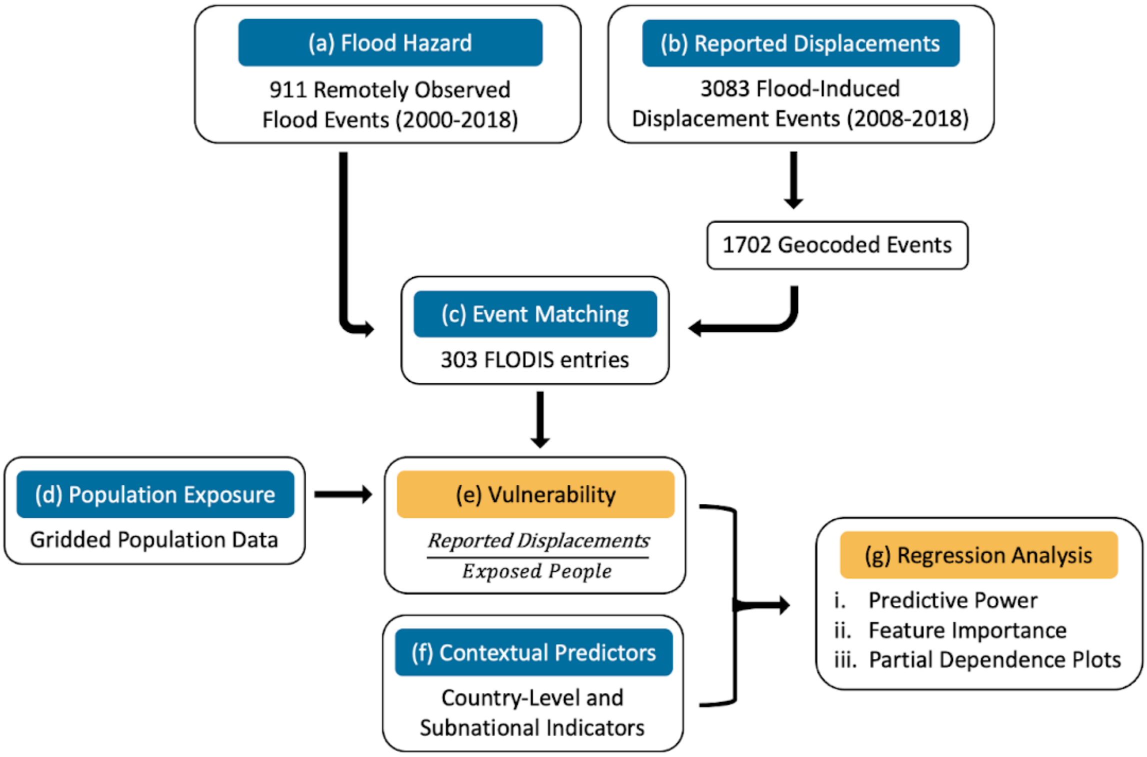

Figure S17. Schematic overview of the data used in the regression analysis. 913 floods are available in the GFD **(a)**; 3083 flood-induced displacement events of the GIDD give information about the number of displacements **(b)**. After geocoding the GIDD, events of both databases are matched in space and time (Mester et al., 2022) **(c)**, yielding 297 matches. For these 297 floods, gridded population data is used to calculate flood exposure **(d)** which is then combined with the reported displacements to calculate flood-specific vulnerability **(e)**. Country-level and subnational predictors are assigned to each event **(f)**. In the regression analysis, the most important predictors and their influence on vulnerability are being assessed **(g)**.

Supplementary Tables

Table S1. As Table 1 in the main paper but for models ranked by AIC.

Predictor 1	Predictor 2	Predictor 3	AIC
Log(GDPpc)	log(Elevation)	Log(Population Density)	944.2
Event Duration	log(Elevation)	Log(Population Density)	948.9
log(Elevation)	Log(Population Density)	-	953.5
log(Elevation)	Population ≤ 14 years	Log(Population Density)	955.7
log(Elevation)	FLOPROS	Log(Population Density)	958.6

Predictor 1	Predictor 2	Predictor 3	AIC
Mortality rate, infant (per 1,000 live births)	Educational index	Urban population (% of total population)	204.7
Population density (people per sq. km of land ...	Educational index	Urban population (% of total population)	207.5
Educational index	Urban population (% of total population)	-	208.1
GDP per capita, PPP (current international $)	Educational index	Urban population (% of total population)	208.9
Mortality rate, infant (per 1,000 live births)	Urban population (% of total population)	-	209.0

Table S2. As Table 1 in the main paper but for models ranked by BIC.

Predictor 1	Predictor 2	Predictor 3	BIC
Log(GDPpc)	log(Elevation)	Log(Population Density)	958.9

Event Duration	log(Elevation)	Log(Population Den-sity)	963.6
log(Elevation)	Log(Population Den-sity)	-	964.6
log(Elevation)	Population ≤ 14 years	Log(Population Den-sity)	970.5
log(Elevation)	FLOPROS	Log(Population Den-sity)	973.4

Predictor 1	Predictor 2	Predictor 3	BIC
Mortality rate, infant (per 1,000 live births)	Educational index	Urban population (% of total population)	213.8
Educational index	Urban population (% of total population)	-	215.0
Mortality rate, infant (per 1,000 live births)	Urban population (% of total population)	-	215.8
Population density (people per sq. km of land ...	Educational index	Urban population (% of total population)	216.6
GDP per capita, PPP (current inter-national $)	Educational index	Urban population (% of total population)	218.0

Table S3. Descriptive statistics for event-level analysis (top) and country-level analysis (bottom).

	count	mean	std	min	25%	50%	75%	max
**Event duration**	297.0	18.757	18.192	1.000	7.000	13.000	26.500	111.000
**log(Population density)**	297.0	2.306	0.884	-0.409	1.710	2.369	2.935	4.743
**log(Total critical infrastructure)**	297.0	-2.094	0.542	-5.000	-2.412	-2.040	-1.679	-0.822
**log(Urban area)**	297.0	-2.385	0.603	-4.222	-2.738	-2.247	-2.005	-0.843
**log(Elevation)**	297.0	2.174	0.792	0.194	1.674	2.178	2.793	3.674
**log(Slope)**	297.0	-0.075	0.492	-1.309	-0.462	-0.122	0.310	1.257
**Population ≤ 14 years**	297.0	0.310	0.131	0.000	0.233	0.317	0.420	0.527
**Population ≥ 65 years**	297.0	0.064	0.052	0.000	0.035	0.051	0.077	0.288
**FLOPROS**	297.0	39.545	72.767	0.000	6.000	15.838	49.978	466.667
**log(GDP per capita)**	297.0	3.780	0.428	2.683	3.499	3.791	4.034	5.004
**Dummy**	297.0	0.479	0.288	0.003	0.235	0.495	0.721	0.997

	count	mean	std	min	25%	50%	75%	max
**log(Population density)**	72.0	1.734	0.581	0.309	1.375	1.796	2.120	3.084
**log(GDP per capita)**	72.0	3.871	0.491	2.829	3.497	3.963	4.209	4.764
**Infant mortality rate**	72.0	28.539	22.432	2.800	9.275	24.400	41.300	87.500
**Population ≤ 14 years**	72.0	31.532	10.832	13.451	22.995	29.433	42.486	50.165
**Population ≥ 65 years**	72.0	7.164	5.397	1.941	2.946	5.273	8.380	21.655
**Population growth**	72.0	1.618	1.196	-0.893	0.798	1.550	2.652	5.167
**Urban population growth**	72.0	2.504	1.616	-0.401	1.301	2.305	3.827	6.228
**Urban population**	72.0	52.761	23.522	13.032	32.709	52.830	74.403	95.045
**Electoral Democracy Index**	72.0	0.506	0.236	0.082	0.300	0.511	0.673	0.894
**Educational Index**	72.0	0.613	0.183	0.214	0.475	0.643	0.730	0.942
**Dummy**	72.0	0.459	0.313	0.008	0.191	0.396	0.735	0.999

References

GADM. (2018). *Database of Global Administrative Areas*. https://gadm.org/data.html
